# Supplementary material for: Host-Parasite Interaction in Sarcoptes scabiei Infestation in Porcine Model with a Preliminary Note on Its Genetic Lineage from India
Source: Animals (Basel). 2020 Dec 7;10(12):2312. doi: 10.3390/ani10122312 (PMC7762329; doi:10.3390/ani10122312)
Supplement: Supplementary file 1 [file animals-10-02312-s001.pdf]

Supplementary Materials: Host-Parasite Interaction in *Sarcoptes scabiei* Infestation in Porcine Model with a Preliminary Note on its Genetic Lineage from India

Arun Kumar De, Sneha Sawhney, Samiran Mondal, Perumal Ponraj, Sanjay Kumar Ravi, Gopal Sarkar, Santanu Banik, Dhruba Malakar, Kangayan Muniswamy, Ashish Kumar, Arvind Kumar Tripathi, Asit Kumar Bera and Debasis Bhattacharya

Table S1. COX1 and VSSC sequences used in the study.

S. No.	Host	Accession No.	Country	Source
		COX1		
1.	Pig	MN986998	Andaman, India	Our sequence
2.	Pig	MN986997	Andaman, India	Our sequence
3.	Human	KR477867	HongKong	GenBank
4.	Human	KR477866	HongKong	GenBank
5.	Human	MK609481	South Korea	GenBank
6.	Human	MK609480	South Korea	GenBank
7.	Human	KR058193	France	GenBank
8.	Human	KR058192	France	GenBank
9.	Human	AY493390	Australia	GenBank
10.	Human	AY493389	Australia	GenBank
11.	Raccoon Dog	AB821012	Japan	GenBank
12.	Dog	AB821011	Japan	GenBank
13.	Serow	AB821010	Japan	GenBank
14.	Marten	AB821009	Japan	GenBank
15.	Human	KJ748526	China	GenBank
16.	Human	KJ748522	China	GenBank
17.	Vulpes	KP987794	Israel	GenBank
18.	Erinaceus	KP987793	Israel	GenBank
19.	Golden Jackal	KP987792	Isreal	GenBank
20.	Oryctolagus	KP987791	Isreal	GenBank
21.	Pig	KP987789	Isreal	GenBank
22.	Sheep	AB779611	Egypt	GenBank
23.	Cattle	AB779607	Egypt	GenBank
24.	Rabbit	AB779606	Egypt	GenBank
25.	Human	AY493381	Panama	GenBank
26.	Human	AY493380	Panama	GenBank
27.	Fox	KT961031	France	GenBank
28.	Koala	MF083743	Australia	GenBank
29.	Wombat	MF083741	Australia	GenBank
30.	Wallaby	AY493398	Australia	GenBank
31.	Dog	KT961028	China	GenBank
32.	Dog	KT961027	Thailand	GenBank
33.	Dog	KT961026	South Africa	GenBank
34.	Dog	KT961025	Italy	GenBank
35.	Dog	KT961029	France	GenBank
36.	Pig	MN985652	USA	GenBank
37.	Dog	JXLN01000001	USA	GenBank

38.	Chimp	AY493396	Tanzania	GenBank
39.	Rabbit	EU256389	China	GenBank
40.	Pig	EU256387	China	GenBank
		**VSSC**		
41.	Pig	MN986999	Andaman, India	Our sequence
42.	Pig	KP974702	Israel	GenBank
43.	Pig	KP974703	Israel	GenBank
44.	Pig	KP974704	Israel	GenBank
45.	Pig	DQ077150	Australia	GenBank
46.	Pig	DQ145115	Australia	GenBank
47.	Golden Jackal	KP974695	Israel	GenBank
48.	Golden Jackal	KP974696	Israel	GenBank
49.	Golden Jackal	KP974697	Israel	GenBank
50.	Erinaceus	KP974698	Israel	GenBank
51.	Oryctolagus	KP974699	Israel	GenBank
52.	Oryctolagus	KP974700	Israel	GenBank
53.	Oryctolagus	KP974701	Israel	GenBank
54.	Vulpes	KP974705	Israel	GenBank
55.	Human	DQ077149	Australia	GenBank
56.	Canis	EF041516	Australia	GenBank

Table S2. Reference values of different biochemical parameters in pigs.

Sl No.	Parameters	Unit	Range	Reference
1.	Total Protein	g/dL	7.9–8.9	[1]
2.	Albumin	g/dL	1.9–3.9	[1]
3.	Globulin	g/dL	5.3–6.4	[1]
4.	Glucose	mg/dL	85–150	[1]
5.	AST	mg/dL	32–84	[1]
6.	ALP	U/L	118–395	[1]
7.	ALT	U/L	37.72–92.81	Rispet et al., 1993[2]
8.	CK	U/L	104.79–5987.43	Rispet et al., 1993[2]
9.	LDH	U/L	461.86–1706	Rispet et al., 1993[2]
10.	TC	mg/dL	78.88–84.68	Kozera et al., 2016[3]
11.	TG	mg/dL	23.02–30.99	Kozera et al., 2016[3]
12.	HDL	mg/dL	38.67–40.60	Kozera et al., 2016[3]
13.	LDL	mg/dL	34.41–39.05	Kozera et al., 2016[3]
13.	Cortisol	ng/ml	27.50–31.80	Radostits et al., 2000[4]

References

1. MERCK MANUAL Veterinary Manual. Available online: https://www.merckvetmanual.com/multimedia/table/v3363365# (accessed on 27th November, 2020)
2. Rispat, G.; Slaoui, M.; Weber, D.; Salemink, P.; Berthoux, C.; Shrivastava, R. *Lab. Anim.* **1993**, 27, 368–373. doi: 10.1258/002367793780745561.
3. Kozera, W.J.; Karpiesiuk, K.; Bugnacka, D.; Falkowski, J.; Milewska, W. *S. Afr. J. Anim. Sci.* **2016**, 46, http://dx.doi.org/10.4314/sajas.v46i1.9.
4. Radostits, O.M.; Gay, C.C.; Blood, D.C.; Hinchcliffe, K.W. Appendix 3 Laboratory Reference Values: Biochemistry Clinical Examination of Farm Animals. In *Veterinary Medicine A Textbook of the Diseases of Cattle, Sheep, Pigs, Goats and Horses*, 9th ed.; Peter, G.G.J., Peter, D.C., Eds.; W.B. Saunders Ltd.: London UK, 2000; Volume, pp. 1819–1822.

Publisher's Note: MDPI stays neutral with regard to jurisdictional claims in published maps and institutional affiliations.

© 2020 by the authors. Licensee MDPI, Basel, Switzerland. This article is an open access article distributed under the terms and conditions of the Creative Commons Attribution (CC BY) license (http://creativecommons.org/licenses/by/4.0/).
